# Development of Loop-Mediated Isothermal Amplification (LAMP) Assays for Rapid Detection of *Ehrlichia ruminantium*

**DOI:** 10.1186/1471-2180-10-296

**Published:** 2010-11-19

**Authors:** Ryo Nakao, Ellen Y Stromdahl, Joseph W Magona, Bonto Faburay, Boniface Namangala, Imna Malele, Noboru Inoue, Dirk Geysen, Kiichi Kajino, Frans Jongejan, Chihiro Sugimoto

**Affiliations:** 1 Department of Collaboration and Education, Research Center for Zoonosis Control, Hokkaido University, Kita 20, Nishi 10, Kita-ku, Sapporo, Hokkaido 001-0020, Japan; 2 Entomological Sciences Program, U.S. Army Public Health Command (Provisional), Aberdeen Proving Ground, Maryland 21010-5403, USA; 3 National Livestock Resources Research Institute (NaLIRRI), P.O. Box 96, Tororo, Uganda; 4 International Trypanotolerance Centre, PMB 14, Banjul, The Gambia; 5 Department of Paraclinical Studies, School of Veterinary Medicine, University of Zambia, P.O. Box 32379, Lusaka, Zambia; 6 Tsetse & Trypanosomiasis Research Institute, P.O. Box 1026, Tanga, Tanzania; 7 National Research Center for Protozoan Diseases, Obihiro University of Agriculture and Veterinary Medicine, Nishi 2-13, Inada-cho, Obihiro, Hokkaido 080-8555, Japan; 8 Prince Leopold Institute of Tropical Medicine, Nationalestraat 155, B-2000 Antwerp, Belgium; 9 Utrecht Centre for Tick-borne Diseases (UCTD), Department of Infectious Diseases and Immunology, Faculty of Veterinary Medicine, Utrecht University, Yalelaan 1, 3584CL, Utrecht, The Netherlands; 10 Department of Veterinary Tropical Diseases, Faculty of Veterinary Science, University of Pretoria, Private Bag X04, 0110, Onderstepoort, South Africa; 11 Department of Diagnostic Medicine/Pathobiology, College of Veterinary Medicine, Kansas State University, Manhattan, KS 66506, USA

## Abstract

**Background:**

The rickettsial bacterium *Ehrlichia ruminantium *is the causative agent of heartwater, a potential zoonotic disease of ruminants transmitted by ticks of the genus *Amblyomma*. The disease is distributed in nearly all of sub-Saharan Africa and some islands of the Caribbean, from where it threatens the American mainland. This report describes the development of two different loop-mediated isothermal amplification (LAMP) assays for sensitive and specific detection of *E. ruminantium*.

**Results:**

Two sets of LAMP primers were designed from the pCS20 and *sodB *genes. The detection limits for each assay were 10 copies for pCS20 and 5 copies for *sodB*, which is at least 10 times higher than that of the conventional pCS20 PCR assay. DNA amplification was completed within 60 min. The assays detected 16 different isolates of *E. ruminantium *from geographically distinct countries as well as two attenuated vaccine isolates. No cross-reaction was observed with genetically related Rickettsiales, including zoonotic *Ehrlichia *species from the USA. LAMP detected more positive samples than conventional PCR but less than real-time PCR, when tested with field samples collected in sub-Saharan countries.

**Conclusions:**

Due to its simplicity and specificity, LAMP has the potential for use in resource-poor settings and also for active screening of *E. ruminantium* in both heartwater-endemic areas and regions that are at risk of contracting the disease.

## Background

The rickettsial bacterium *Ehrlichia ruminantium *is a causative agent of heartwater, the disease of ruminants transmitted by ticks of the genus *Amblyomma *[1]. Heartwater is not only responsible for high economic losses in endemic countries [2], but is also suggested to be a potential emerging zoonosis since the PCR and sequence detection of the pathogen's presence in three fatal human cases although the cytological examination and bacterial isolation were not achieved [3,4]. The disease is established in nearly all countries of sub-Saharan Africa and some islands of the Caribbean, from where it threatens the American mainland [5]. In the USA, three *Ehrlichia *species, namely *E. canis*, *E. chaffeensis*, and *E. ewingii*, are known to exist [6-11]. Recently, Panola Mountain (PM) *Ehrlichia*, which is closely related to *E. ruminantium*, was discovered as a novel zoonotic *Ehrlichia *in the state of Georgia [12,13]. Active surveillance using a reliable method which can discriminate *E. ruminantium *from these other *Ehrlichia *species is an asset in preventing introduction of heartwater into the USA.

In heartwater endemic countries, conventional diagnosis is based upon clinical signs and microscopic examination of post-mortem brain smears. As a more reliable and sensitive diagnostic method, several PCR-based assays have been developed for the detection of *E. ruminantium*, including conventional PCR [14-16], nested PCR [17,18], and real-time PCR [19,20]. Among them, the pCS20 real-time PCR TaqMan probe assay provides the best sensitivity with a detection limit of one gene copy per reaction, which is 100 times higher than that of conventional pCS20 PCR [20]. However, this assay was reported to cross-react with both *E. chaffeensis *and *E. canis *[20]. Moreover, although this assay performs well in the sensitive detection and quantification of *E. ruminantium*, it is not readily transferable to low-technology settings where there is limited access to expensive fluorescence detector based thermocyclers.

Loop-mediated isothermal amplification (LAMP) assay is a rapid DNA amplification method originally developed by Notomi et al. [21], and it has been applied for the detection of viral [22,23], bacterial [24,25], fungal [26], and parasitic agents [27,28], but it has never previously been applied to rickettsial agents. The method requires a specially designed primer set that recognizes at least six independent regions of the target gene, which increases the specificity as well as the rapidity of the reaction. LAMP results are visualized by turbidity that can be seen by the naked eye [29], and optionally by agarose gel electrophoresis or by addition of fluorescent dyes visualized under UV light [30,31]. Since the *Bst *DNA polymerase used in LAMP allows strand displacement-DNA synthesis, LAMP reactions are performed under isothermal conditions using a simple incubator, such as a water bath or heating block. Furthermore, LAMP reagents are relatively stable for a month, even when stored at 37°C, which is a warmer temperature than recommended by the manufacturer [32]. With these advantages, LAMP has the potential to be used even in clinical laboratories often poorly equipped, facing problems of constant electricity supply in tropical and sub-tropical countries where heartwater is endemic.

The purpose of the present study was to develop LAMP assays for the detection of *E. ruminantium *and to evaluate the diagnostic sensitivity and specificity of these assays using a panel of bacterial DNA samples, quantitated plasmid standards, and field samples derived from both animal blood and ticks. The newly developed LAMP assays successfully detected *E. ruminantium *with rapidity, specificity, and high sensitivity.

## Results

### Optimization of LAMP

The reactions for both pCS20 and *sodB *LAMP were performed under isothermal conditions at a range of 58 to 66°C using plasmid DNA (10^6 ^copies per reaction) for 120 min, with monitoring of the turbidity. Although amplifications with the LAMP assays were observed at all temperatures tested, the reactions reached the threshold value (0.1) with the shortest incubation times at 61°C for pCS20 and 63°C for *sodB *(data not shown). No nonspecific amplification was detected for the negative cell culture until after at least 120 min incubation. Thus, subsequent LAMP reactions were conducted at these temperatures for 60 min.

### Sensitivity of LAMP assays

**Figure 1:**
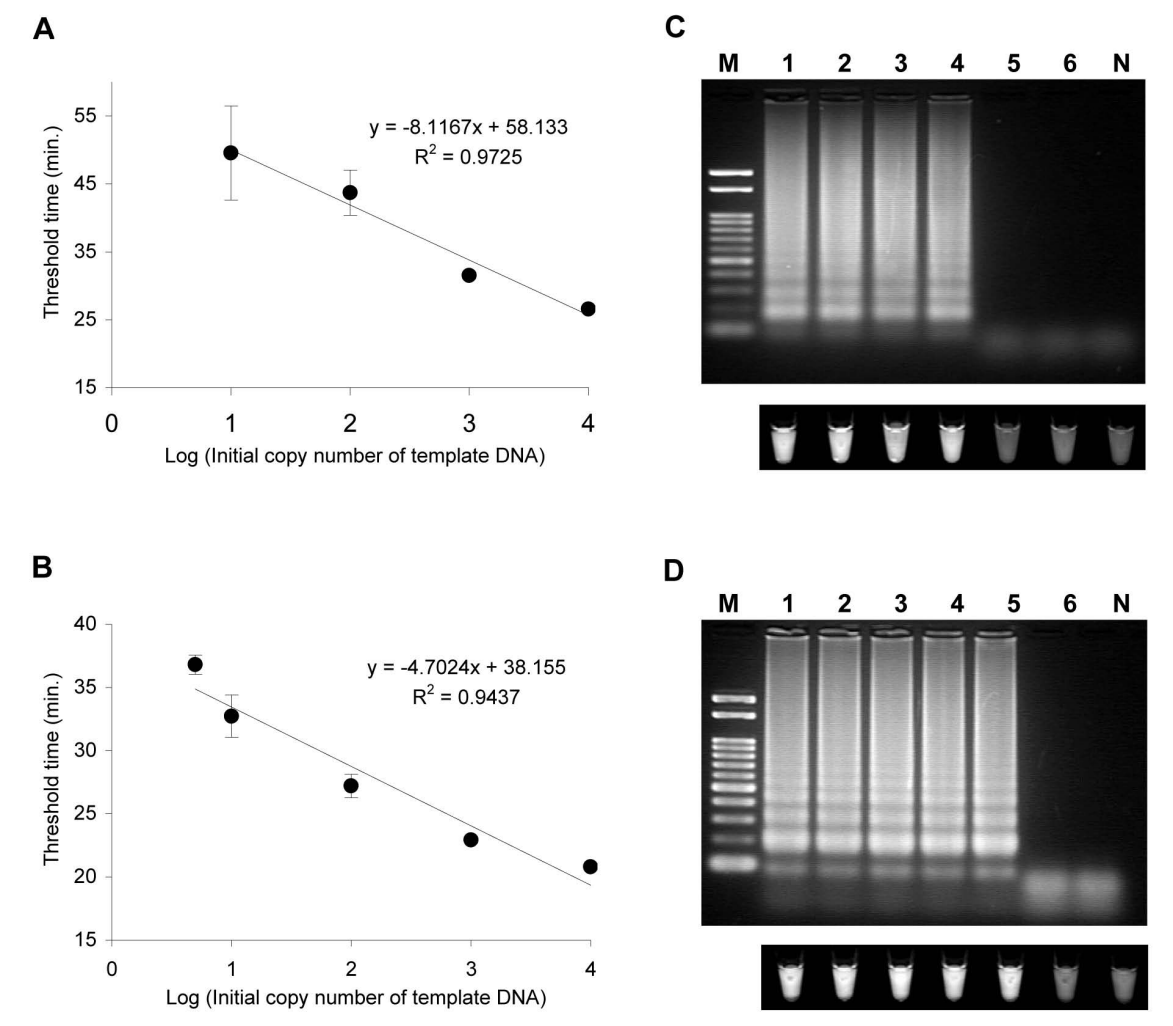
**Sensitivities of *E. ruminantium *LAMP assays**. The assays were performed with serial dilutions of plasmid DNA (10^4^, 10^3^, 10^2^, 10, 5, and 1 copies per reaction) containing the pCS20 or *sodB *genes. (A and B) Real-time monitoring of pCS20 (A) and *sodB *(B) LAMP assays using the Loopamp real-time turbidimeter. Plots represent the mean threshold time (Turbidity of 0.1). The error bars represent the standard errors of the mean from three replicates. The plot of the mean threshold time versus the log of the input DNA fit a linear function (R^2 ^= 0.9725 for pCS20 LAMP and 0.9437 for *sodB *LAMP). (C and D) Visual detection of pCS20 (C) and *sodB *(D) LAMP products. LAMP products were visualized with Gel-Red TM under UV (lower panel) or electrophoresed in a 2.0% agarose gel stained with Gel-Red TM (upper panel). Lanes: M, 100-bp molecular weight marker; 1 to 6, from left to right, 10^4 ^to 1 gene copy per reaction, as above; N, negative control.

The sensitivities of pCS20 and *sodB *LAMP assays are shown in Figure 1A, and 1B, respectively. A plot of the threshold time versus the log of the initial template copy number showed a linear regression, with statistically significant regression coefficients (R^2 ^= 0.9725 for pCS20 and 0.9473 for *sodB *LAMP). The detection limits for these assays, using a positive turbidity signal as the indicator, were 10 copies for pCS20 and 5 copies for *sodB *LAMP. Alternative detection methods included agarose gel electrophoresis of the LAMP products, which displayed the typical ladder-like pattern (Figure 1C and 1D, upper panels), as well as the detection of double stranded LAMP products using Gel-Red (Figure 1C and 1D, lower panels). With smaller amounts of DNA in triplicate assays, 5 copies of pCS20 was amplified once, with a threshold time of 48.3 min, and 1 copy of *sodB *was amplified twice with threshold times of 45.7 and 49.4 min.

### Specificity of LAMP assays

The specificity of pCS20 and *sodB *LAMP assays was evaluated by using the genomic DNA of 18 known *E. ruminantium *isolates and five closely related species of Anaplasmataceae: *E. canis*, *E. chaffeensis*, *Anaplasma centrale*, *A. marginale*, and *A. phagocytophilum*. All isolates of *E. ruminantium *were positive in both LAMP assays, the pCS20 real-time PCR and the pCS20 PCR; whereas the pCS20 PCR was cross-reactive with both *E. canis *and *E. chaffeensis *(Table 1).

**Table 1:** Specificities of pCS20 PCR, pCS20 real-time PCR, pCS20 LAMP, and *sodB *LAMP assays

Rickettsial bacteria	Isolate	Origin	pCS20 PCR	pCS20 real-time PCR	pCS20 LAMP	*sodB *LAMP
*Ehrlichia ruminantium*	Ball 3	South Africa	+	+	+	+
	Burkina Faso	Burkina Faso	+	+	+	+
	Crystal Springs	Zimbabwe	+	+	+	+
	Gardel	Guadelope, Caribbean	+	+	+	+
	attenuated Gardel	Guadelope, Caribbean	+	+	+	+
	Ifé Nigeria	Nigeria	+	+	+	+
	Kerr Seringe	Gambia	+	+	+	+
	Kiswani	Kenya	+	+	+	+
	Kwanyanga	South Africa	+	+	+	+
	Lutale	Zambia	+	+	+	+
	Pokoase 471	Ghana	+	+	+	+
	Sankat 430	Ghana	+	+	+	+
	São Tomé	São Tomé and Principe	+	+	+	+
	Senegal	Senegal	+	+	+	+
	attenuated Senegal	Senegal	+	+	+	+
	Um Banein	Sudan	+	+	+	+
	Welgevonden	South Africa	+	+	+	+
	Zeerust	South Africa	+	+	+	+
*Ehrlichia canis*			+	-	-	-
*Ehrlichia chaffeensis*			+	-	-	-
*Anaplasma centrale*			-	-	-	-
*Anaplasma marginale*			-	-	-	-
*Anaplasma phagocytophilum*			-	-	-	-

### Inhibitory effect of DNA preparation purified from bovine blood or ticks

In order to access inhibitory effects of components present in field samples, mixtures of standard plasmid DNA and DNA extracts from bovine blood and *Amblyomma variegatum *were tested by the LAMP assays. When DNA extracts from bovine blood were added to the templates, both pCS20 and *sodB *LAMP could not detect 10 copies in two samples, which is in accordance with real-time PCR (Table 2). When DNA extracts from *A. variegatum *were added to the templates, both pCS20 and *sodB *LAMP failed in detecting 10 copies in all five samples, while real-time PCR could detect in four. The pCS20 PCR using the KAPA Blood PCR kit detected more positives than the pCS20 PCR using the AmpliTaq Gold PCR kit in the templates with 10^2 ^and 10^3 ^copies (Table 2).

**Table 2:** Inhibitory effects of DNA extracts from field samples on pCS20 PCR, pCS20 real-time PCR, pCS20 LAMP, and *sodB *LAMP

		No. of samples:				
		
Sample type	No. of plasmid copies per reaction	Tested	pCS20 PCR positive	pCS20 real-time PCR positive	pCS20 LAMP positive	*sodB *LAMP positive
DNA extracts from bovine blood	1	5	0 (0)^a^	0	0	0
	10	5	0 (0)	3	3	3
	10^2^	5	2 (0)	5	4	5
	10^3^	5	5 (0)	5	5	5
	10^4^	5	5 (5)	5	5	5
DNA extracts from *Amblyomma variegatum*	1	5	0 (0)	0	0	0
	10	5	0 (0)	4	0	0
	10^2^	5	5 (0)	5	5	5
	10^3^	5	5 (3)	5	5	5
	10^4^	5	5 (5)	5	5	5

^a^Total no. of samples positive by using the KAPA Blood PCR kit (Total no. of samples positive by using the AmpliTaq Gold PCR kit).

### Detection of *E. ruminantium *DNA in field samples

A total of 140 *A. variegatum *ticks were collected in 7 sites in Uganda and individually analyzed for the presence of *E. ruminantium *DNA. Out of 140 ticks, including 96 males and 44 females, 12 ticks (11 male and 1 female) were found positive with both pCS20 LAMP and *sodB *LAMP. The pCS20 real-time PCR detected 13 positives, including the 12 LAMP-positive ticks and an additional tick from Dokolo, while pCS20 PCR could detect only 8 positives (Table 3). All the samples found positive with PCR were also positive with LAMP. The percent positive with LAMP (8.57%) was higher than with PCR (5.71%) but slightly lower than with real-time PCR (9.29%). Of the 150 bovine, 35 goat, and 19 lamb blood samples analyzed, two lamb samples were positive using PCR, real-time PCR, and LAMP (Table 3).

**Table 3:** Comparison of pCS20 PCR, pCS20 real-time PCR, pCS20 LAMP, and *sodB *LAMP for the detection of *E. ruminantium *in various field samples

		No. of samples:				
		
Sample type	Origin (Site/Country)	Tested	pCS20 PCR^a ^positive	pCS20 real-time PCR positive	pCS20 LAMP positive	*sodB *LAMP positive
Bovine blood	Butaleja/Uganda	50	0	ND^b^	0	0
	Petauke/Zambia	50	0	ND	0	0
	Serengeti/Tanzania	50	0	ND	0	0

Goat blood	Chama/Zambia	35	0	ND	0	0

Lamb's blood	Kerr Seringe/The Gambia	19	2	2	2	2

Sheep blood^c^	NA^d^	4	4	4	4	4

Tick;*Amblyomma variegatum*	Amuria/Uganda	20 (15/5)^e^	2 (2/0)	4 (4/0)	4 (4/0)	4 (4/0)
	Butaleja/Uganda	20 (18/2)	0	1 (1/0)	1 (1/0)	1 (1/0)
	Dokolo/Uganda	20 (12/8)	1 (1/0)	2 (2/0)	1 (1/0)	1 (1/0)
	Kaberamaido/Uganda	20 (14/6)	0	1 (0/1)	1 (0/1)	1 (0/1)
	Pallisa/Uganda	20 (10/10)	2 (2/0)	2 (2/0)	2 (2/0)	2 (2/0)
	Soroti/Uganda	20 (17/3)	2 (2/0)	2 (2/0)	2 (2/0)	2 (2/0)
	Tororo/Uganda	20 (10/10)	1 (1/0)	1 (1/0)	1 (1/0)	1 (1/0)
	
	Subtotal for tick samples	140 (96/44)	8 (8/0)	13 (12/1)	12 (11/1)	12 (11/1)

^a^PCR was performed using KAPA Blood PCR kit.

^b^ND, not done.

^c^Blood samples from sheep experimentally infected with *E. ruminantium *were used as positive controls.

^d^NA, not applicable.

^e^Total no. of ticks (No. of male ticks/No. of female ticks).

### Cross-reactivity of LAMP with zoonotic *Ehrlichia *in the USA

LAMP assays were conducted with 17 *Amblyomma americanum *DNA samples from the USA that had previously tested positive for *E. chaffeensis*, *E. ewingii*, or PM *Ehrlichia *(Table 4). Both of the genetic clades of PM *Ehrlichia *that have been described were represented among these samples. All 17 samples tested negative using both LAMP assays (data not shown).

**Table 4:** Collection details for 17 *A. americanum *from the USA harboring DNA from *Ehrlichia *species

*Ehrlichia *detected^a^	MAP1 types^b^	Co-infection with other *Ehrlichia*	Patient	Tick isolation site
Panola Mountain *Ehrlichia*	Clade 2		22-year-old female	Kentucky
	B180/PMtn		52-year-old male	Maryland
	B180/PMtn		25-year-old male	Maryland
	Unknown	*Ehrlichia ewingii*	50-year-old male	Maryland
	Clade 2	*Ehrlichia chaffeensis*	41-year-old male	New Jersey
	PME + Clade 2		46-year-old male	New Jersey
	B180/PMtn		41-year-old male	New Jersey
	B180/PMtn		31-year-old male	New Jersey
	B180/PMtn		46-year-old male	New Jersey
	B180/PMtn		NR^c^	Oklahoma
	Unknown		25-year-old male	Virginia
*Ehrlichia chaffeensis*			29-year-old male	Virginia
			18-year-old female	South Carolina
*Ehrlichia ewingii*			Male^d^	Virginia
			Male	Virginia
			36-year-old male	Virginia
			34-year-old male	Virginia

^a^*Ehrlichia *species were detected by previously described assays [42,45].

^b^MAP1 types; B180, Clade 2, PME, and PMtn, represents the phylogenetic clade based on the sequence of Major Antigenic Protein 1 (MAP1) gene [42].

^c^NR, not recorded.

^d^Age was not recorded.

## Discussion

This report describes the development of two *E. ruminantium*-specific LAMP assays based on the pCS20 and *sodB *genes. The pCS20 region was the first target used for the genetic detection of *E. ruminantium *[33]. Subsequently, Peter et al. developed a PCR assay targeting pCS20 region with primers AB128 and AB129 for sensitive and specific detection of *E. ruminantium *[14]. This assay was further evaluated for its reliability by the same authors [15] and has been widely used by many researchers [12,17,18,34]. Because primers AB128/129 could not amplify the divergent isolate of Kümm2, van Heerden et al. designed primers HH1F and HH2R in a highly conserved region of pCS20 [16]. However, the major drawback of latter assay was cross-reactivity with closely related bacteria such as *E. canis *and *E. chaffeensis*, which were not detected by former assay [14,15]. Although pCS20 real-time PCR was also reported to be cross-reactive with *E. canis *and *E. chaffeensis *[20], our study did not give the same results (Table 1). This inconsistency may be explained by the differences of sequence in pCS20 region between isolates as observed in *E. ruminantium *[16]. Thus, in this study, we have developed LAMP assays based on not only pCS20 but also *sodB *because of its high degree of conservation among isolates.

**Figure 2:**
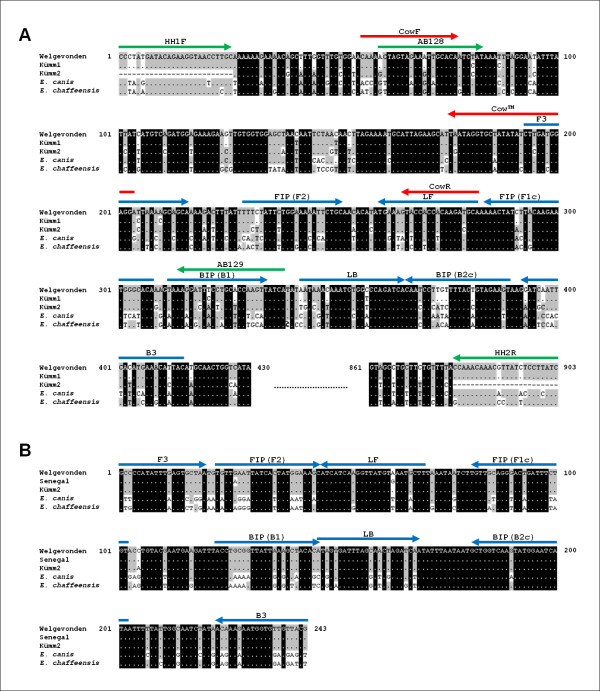
**Nucleotide sequence alignment of the target regions of pCS20 (A) and *sodB *(B) genes**. The locations of the primer recognition sites are indicated by arrows, together with the primer names. The blue, green and red arrows represent primers for the LAMP, conventional PCR, and real-time PCR, respectively.

The pairwise sequence identities calculated for pCS20 showed that the lowest pairwise identity for pCS20 sequences was 83.95% (between Kümm1 and Kümm2 isolates), whereas that the lowest pairwise identity for the more conserved *sodB *gene was 99.00% (between Senegal and Kümm2 isolates) [35]. This implies that *sodB *might be a more suitable target than pCS20 for the genetic detection of this species. Compared to the sequence of Welgevonden isolate, the Kümm2 differs by 24 out of 187 bp in the region targeted by the pCS20 LAMP, while there is no sequence difference in the region targeted by *sodB *LAMP (Figure 2). Although both pCS20 and *sodB *LAMP detected all the *E. ruminantium *isolates tested in the present study, *sodB *LAMP is more likely to detect previously unknown, divergent isolates of *E. ruminantium*. Thus, we concluded that *sodB *LAMP is more suitable for detecting *E. ruminantium *and the diagnosis will be made more reliable in combination with pCS20 LAMP.

The detection limits of the pCS20 and *sodB *LAMP assays were 10 and 5 copies per reaction, respectively, which are at least 10-times more sensitive than that of conventional pCS20 PCR but slightly less sensitive than pCS20 real-time PCR [20]. According to the instructions for LAMP primer design software, the stability of primer end, especially 5' end of F1c/B1c and 3' end of F2/B2 as well as F3/B3, is one of the crucial factors for designing proper LAMP primers http://loopamp.eiken.co.jp/e/lamp/primer.html. When LAMP primers were designed for conserved pCS20 regions within isolates, only limited number of primer candidates were obtained initially (data not shown). Therefore, we had to change the optimal values of parameters in the software for further designing pCS20 LAMP primers. In fact, an index for stability of primer, the dG value of the 5' end of the pCS20 B1c region (-3.69 kcal/mol), is above the value recommended by manufactures (< -4.00 kcal/mol), which may explain lower sensitivities of pCS20 LAMP than *sodB *LAMP.

As is documented in several reports [24,36], LAMP showed relative tolerance to PCR inhibitors in blood, which was comparable to pCS20 real-time PCR (Table 2). However, LAMP was clearly inhibited when DNA extracts from *A. variegatum *were included in the reaction (Table 2). It is known that *Amblyomma *tick tissue contains PCR-inhibitory elements which cannot be always removed during DNA purification [14,15]. Thus, LAMP is slightly less sensitive in the presence of such inhibitors in ticks compared to real-time PCR. However, considering that real-time PCR is time-consuming and requires a thermal cycler with real-time monitoring and data analysis systems, which is expensive and can be relatively complicated to use, LAMP has clear advantages over real-time PCR in terms of a practical system in a standard diagnostic laboratory, especially those in developing countries where the disease is prevalent.

In the present study, two sheep blood samples from a heartwater-endemic area tested positive by LAMP (Table 3). Domestic ruminants are known to occasionally harbor *E. ruminantium *without any clinical signs and to serve as reservoirs of the disease after recovery [37]. Previous reports demonstrated that PCR assays could detect the pathogen in the peripheral blood of clinically healthy animals in heartwater endemic areas [20,38], indicating that a DNA-based technique is useful even for the diagnosis of latent infection. Hence, LAMP is a powerful tool not only for the epidemiological study of heartwater but also for the rapid and sensitive diagnosis of infected animals in the disease-endemic areas.

The simplest way of detecting LAMP products is to inspect the white turbidity that results from magnesium pyrophosphate accumulation, as a by-product of the reaction, by naked eye [29]. However, a small amount of this white precipitate is not always distinguishable from other white precipitates, such as proteins or carbohydrates, derived from the templates. As an alternative method, this study employed a closed system, coupled with a double-stranded DNA (dsDNA)-binding dye, for low-cost detection of amplified DNA (Figure 1C and 1D, lower panels). The results obtained by this system were consistent with those obtained by gel electrophoresis (Figure 1C and 1D, upper panels). Since the detection can be accomplished in a closed system, without opening the reaction tubes, the risk of contamination is much lower than in gel electrophoresis or by adding dye at the end of the reaction. Theoretically, it should be possible to replace the Gel-Red TM dye we used with other dyes such as SYBR Green I [22,25,39], ethidium bromide, EvaGreen [30], and PicoGreen [40], which are reported to be useful for the detection of LAMP products.

As well documented by Burridge et al., heartwater may easily be introduced into the American mainland through the introduction of infected ticks or animals from heartwater endemic areas [5]. Once imported, it is likely that the disease could become established because of the presence of local potential tick vectors [5,41]. In order to prevent this pathogen from spreading into the USA, a screening test with high sensitivity and specificity is needed prior to the animal importation. In this respect, the 17 DNA samples from *A. americanum *harboring DNA from *Ehrlichia *species that are enzootic to the USA were found to be negative in LAMP. Considering that the detection limits of the PCR assay used for the detection of *Ehrlichia *species in *A. americanum *were 10 copies per reaction [42], which is comparable to those of LAMP assays, these samples were LAMP-negative not because the DNA concentrations were below the detection levels but probably because there were no cross reactions due to sequence mismatches or deletions in the targeted regions.

## Conclusions

The LAMP assays developed in this study allow rapid, sensitive, and specific detection of *E. ruminantium*. Although LAMP reactions were inhibited in the presence of extracts from blood and ticks, the diagnostic sensitivity of LAMP was higher than that of conventional PCR, when tested with field-collected ticks. Since LAMP requires minimal time and equipment to perform, this technique can potentially be used in resource-poor settings where heartwater is endemic. The lack of cross-reactivity with closely related *Ehrlichia *species enhances its utility for active screening in areas under threat of the introduction of the disease.

## Methods

### Rickettsial bacteria

*E. ruminantium *isolates used in this study were: Ball 3, Burkina Faso, Crystal Springs, Gardel, attenuated Gardel, Ifé Nigeria, Kerr Seringe, Kiswani, Kwanyanga, Lutale, Pokoase 471, Sankat 430, São Tomé, Senegal, attenuated Senegal, Um Banein, Welgevonden, and Zeerust. Attenuated isolates of Gardel and Senegal were obtained by serial passages in mammalian cells as previously described [43]. All were cultured in bovine aorta endothelial (BAE) cells as described previously [44] and subjected to DNA extraction. Cultures of closely related rickettsia, including *E. canis, E. chaffeensis, A. centrale*, *A. marginale*, and *A. phagocytophilum*, were also used for LAMP specificity testing.

### Field samples

From July 2008 to January 2009, adult *A. variegatum *ticks were collected from indigenous cattle in seven districts in Uganda: Amuria, Butaleja, Dokolo, Kaberamaido, Pallisa, Soroti, and Tororo. Ticks were pooled and stored in sealed plastic bags containing silica gel until DNA extraction. Twenty ticks from each site were randomly selected, and a total of 140 (96 males and 44 females) samples were used in the present study. From July 2008 to May 2009, blood samples were collected from clinically healthy cattle or goats in four different sites in sub-Saharan countries. Bovine blood samples were collected in Butaleja district in Uganda, Serengeti district in Tanzania, and Petauke district in Zambia. Goat blood samples were obtained from Chama district in Zambia. The former two sites are endemic for East Coast fever caused by *Theileria parva*, and the latter are endemic for trypanosomiosis. These areas are habitats for *Amblyomma *ticks and lacked adequate tick control programs. In total, 150 bovine blood samples, 50 from each site, and 35 goat blood samples were used in the present study. In addition, this study employed DNA samples extracted from the blood of lambs at Kerr Seringe in the Gambia, where heartwater is endemic. Nineteen samples were randomly selected from those used in the previous study, some of which were positive by pCS20 nested PCR [17]. As positive controls, four blood samples obtained from two sheep experimentally infected with *E. ruminantium *Senegal isolate were used. Blood was collected from each sheep on days 14 and 16 post infections when the animals showed high fever. Research on samples from animals was conducted adhering to guidelines for Care and Use of Laboratory Animals and was approved by the Animal Care and Use Committee of the Utrecht University.

### DNA extraction

DNAs from rickettsia-infected cell cultures were extracted using Nucleospin Tissue kits (Macherey-Nagel, Duren, Germany). *A. variegatum *ticks were washed with 70% ethanol and rinsed twice with distilled water. Tick samples were then homogenized by Micro Smash MS-100R (TOMY, Tokyo, Japan) for 2 min at 2,500 rpm, followed by DNA extraction with DNAzol (Invitrogen, Carlsbad, CA). DNAs from blood were extracted using either the GenTLE kit (Takara, Shiga, Japan) or a DNA isolation kit for mammalian blood (Roche, Mannheim, Germany). All procedures were carried out as described by the manufacturers.

### LAMP primers

Two sets of LAMP primers were designed for the pCS20 and *sodB *genes of *E. ruminantium*. The nucleotide sequence of the Welgevonden isolate of *E. ruminantium *was retrieved from GenBank [GenBank:CR767821] and aligned with the available sequences of other isolates to identify conserved regions, using CLUSTALW software version 1.83 (DNA Data Bank of Japan; http://clustalw.ddbj.nig.ac.jp/top-e.html). A potential target region was selected from the aligned sequences, and four primers, comprising two outer (F3 and B3) and two inner (FIP and BIP) primers, were designed using LAMP primer software PrimerExplorer V4 (http://primerexplorer.jp/elamp4.0.0/index.html; Eiken Chemical Co., Japan). Loop primers (LF and LB) were designed manually. The designed primer sequences are shown in Table 5.

**Table 5:** Primer sets used for LAMP assays in the present study

Target gene	Primer type	Sequence (5' to 3')	Length	Amplicon size with F3+B3
pCS20	F3	CTTGATGGAGGATTAAAAGCA	21	161
	B3	GTAATGTTTCATGTGAATTGATCC	24	
	FIP	TGTGCCCATTCTTGTAAGATAGTTT-TTTCTATTCTGGAAAAATTCTGC	48	
	BIP	TAAAGGATTTCCTGCACCAAGTT-ACTTCTACAGTAAAACAAGGATTG	47	
	LF	TGCATCTTGTGGTGGTACTTTCA	23	
	LB	AATAAACAAATCTGGCCCAGATCA	24	
				
*sodB*	F3	GCCCCATATTTGAGTGCTAA	20	180
	B3	CGTAACAACACCATTCTTTGT	21	
	FIP	ACAGAAATCAGTCCCTGCAACA-TGTTGAATTATCACTATGGAAAGC	46	
	BIP	ACCTGCGGTTATTAAAGCTACACA-TATGATTCCATACTTGACCAGC	46	
	LF	AAGCATTTACATAACCTTGATGAT	24	
	LB	ATAGTGATTTAGCAACTAGATCAA	24	

### LAMP reactions

LAMP was carried out in a 25-μl volume reaction, consisting of 2.5-μl of 10× reaction buffer [200 mM Tris/HCl (pH 8.8), 100 mM KCl, 100 mM (NH_4_)_2_SO_4_, 1% Tween 20], 3.5-μl 10 mM dNTPs, 4.0-μl 5 M betaine (Sigma, St Louis, MI), 1.5-μl 100 mM MgSO_4_, 2.0-μl primer mixture (20 μM each of FIP, BIP, LF, and LB primers, and 2.5 μM each of F3 and B3 primers for the pCS20 LAMP; or 20 μM each of FIP, BIP, and LF primers, and 35 μM of LB primers, and 2.5 μM each of F3 and B3 primers for the *sodB *LAMP), 9.5-μl DDW, 1.0-μl (8 U) *Bst *DNA polymerase (New England Biolabs, Beverly, MA), and 1.0-μl template DNA. To find the optimal reaction temperatures for the two LAMP assays, the reaction mixtures were incubated for 120 min at 58 to 66°C in a Loopamp real-time turbidimeter (LA-200; Teramecs, Kyoto, Japan). For the field samples, LAMP reactions were conducted in a heating block.

### Preparation of plasmid standard

The pCS20 and *sodB *genes of *E. ruminantium *were amplified by PCR using the F3 and B3 primers of each LAMP primer set. PCR was carried out using high-fidelity KOD plus DNA polymerase (Toyobo, Tokyo, Japan) in 25-μl reaction mixture containing 1.0 μM of each primer, 200 μM dNTPs, 1.0 unit of KOD plus DNA polymerase, and genomic DNA from *E. ruminantium*, isolate Welgevonden. Amplification was performed for 25 cycles of 95°C for 15 s, 55°C for 15 s, and 72°C for 1 min, followed by a final extension at 72°C for 2 min. The PCR products were poly-A tailed and then cloned into a pGEM-T vector (Promega, Madison, WI). Each plasmid clone was sequenced on an ABI Prism 3130 genetic analyzer (Applied Biosystems, Foster City, CA) with BigDye Terminator version 1.1 (Applied Biosystems), to confirm identity, and was used as the standard plasmid for determining the specificity of the respective LAMP assay. The concentrations of plasmid DNA were measured with a Quant-iT dsDNA BR and Qubit Fluorometer (Invitrogen, Carlsbad, CA) and the corresponding copy numbers were calculated.

### Assessment of LAMP inhibitors in DNA prepared from blood or ticks

Five bovine blood samples and five individual *A. variegatum *ticks were obtained from heartwater free areas and verified negative for *E. ruminantium *by LAMP. Total DNA was extracted as described above. The concentrations of DNA were 0.40-16.56 ng/μl and 1.97-4.20 ng/μl for those extracted from bovine blood and *A. variegatum*, respectively. The standard plasmid was diluted with DNA solution prepared from bovine blood or *A. variegatum *to give final concentrations of 1, 10, 10^2^, 10^3^, 10^4 ^copies of plasmid DNA per microliter.

### LAMP sensitivity and specificity

The sensitivity of each LAMP assay was assessed using each standard plasmid (10^4^, 10^3^, 10^2^, 10, 5, and 1 copies/reaction) in a Loopamp real-time turbidimeter (Model & Maker). Readings were analyzed by LA-200 version 0.18 software (Teramecs, Kyoto, Japan), and positive real-time reactions were determined by taking into account the time taken for the turbidity value to increase above a predetermined threshold value of 0.1 [29]. To confirm that each LAMP amplified the correct target, the product was electrophoresed in a 2.0% agarose gel stained with Gel-Red TM (Biotium, Hayward, CA) or visualized under UV light, as described below. LAMP specificity assays were conducted using 18 different isolates of *E. ruminantium*, isolates of 5 closely related rickettsial bacteria, and tick DNA samples positive for 3 different species of USA ehrlichiae (described below).

### Detection of LAMP products

In addition to monitoring turbidity and gel electrophoresis, we used a common dsDNA-binding dye for the detection of LAMP products. One microliter of the dsDNA-dye mixture, consisting of 25% (v/v) glycerol and Gel-Red TM (1:50 dilution of a 10,000× stock solution), was put inside the lid of LAMP reaction tubes. To prevent dye mixture from dripping with vapor, the reaction mixture was overlaid with one drop of mineral oil. After the reaction terminated, the tubes were inverted several times, and LAMP products were visualized under UV light.

### pCS20 PCR and pCS20 real-time PCR assays

To compare the specificity and sensitivity of the LAMP, conventional PCR and real-time PCR to amplify the pCS20 gene was conducted using primers HH1F and HH2R [16], and CowF, CowR and Cow™ probe [20], respectively (Figure 2). PCR was performed with either the KAPA Blood PCR kit (Kapabiosystems, Boston, MA) or the AmpliTaq Gold PCR kit (Applied Biosystem). In order to reduce the effect of PCR inhibitors in the templates, the KAPA Blood PCR kit was used for the analysis of field samples. PCR products were electrophoresed in a 1.2% agarose gel stained with Gel-Red TM. The real-time PCR was performed with THUNDERBIRD qPCR Mix (Toyobo, Osaka, Japan) and analyzed on Stratagene Mx3000 QPCR System (Stratagene, La Jolla, CA).

### *A. americanum *samples harbouring DNA from *Ehrlichia *species

This study employed 17 DNA samples from *A. americanum *ticks recovered from people in the USA between 2004 and 2006, in which zoonotic *Ehrlichia *(*E. ewingii*, *E. chaffeensis*, or PM *Ehrlichia*) were detected by conventional PCR for the P28 antigen gene (*E. ewingii*) or nested PCR based on the 16S rRNA gene (*E. chaffeensis*) or citrate synthase gene (PM *Ehrlichia*), as described elsewhere [42,45]. Collection details are shown in Table 4.

## Authors' contributions

RN performed LAMP and PCR assays, conducted data analysis, and draft the manuscript. RN, JWM, BN, IM, NI, and CS carried out field sample collections and DNA extractions. EYS, BF, and DG provided DNA samples from lambs or *A. americanum*. KK, JF, and CS conceived of the study, and participated in its design and coordination and helped to finalize the manuscript. All authors read and approved the final manuscript.
